# Supplementary material for: Preparation and Evaluation of Auxiliary Permeable Microneedle Patch Composed of Polyvinyl Alcohol and Eudragit NM30D Aqueous Dispersion
Source: Pharmaceutics. 2023 Jul 22;15(7):2007. doi: 10.3390/pharmaceutics15072007 (PMC10385563; doi:10.3390/pharmaceutics15072007)
Supplement: Supplementary file 1 [file pharmaceutics-15-02007-s001.zip › pharmaceutics-2473386-supplementary.pdf]

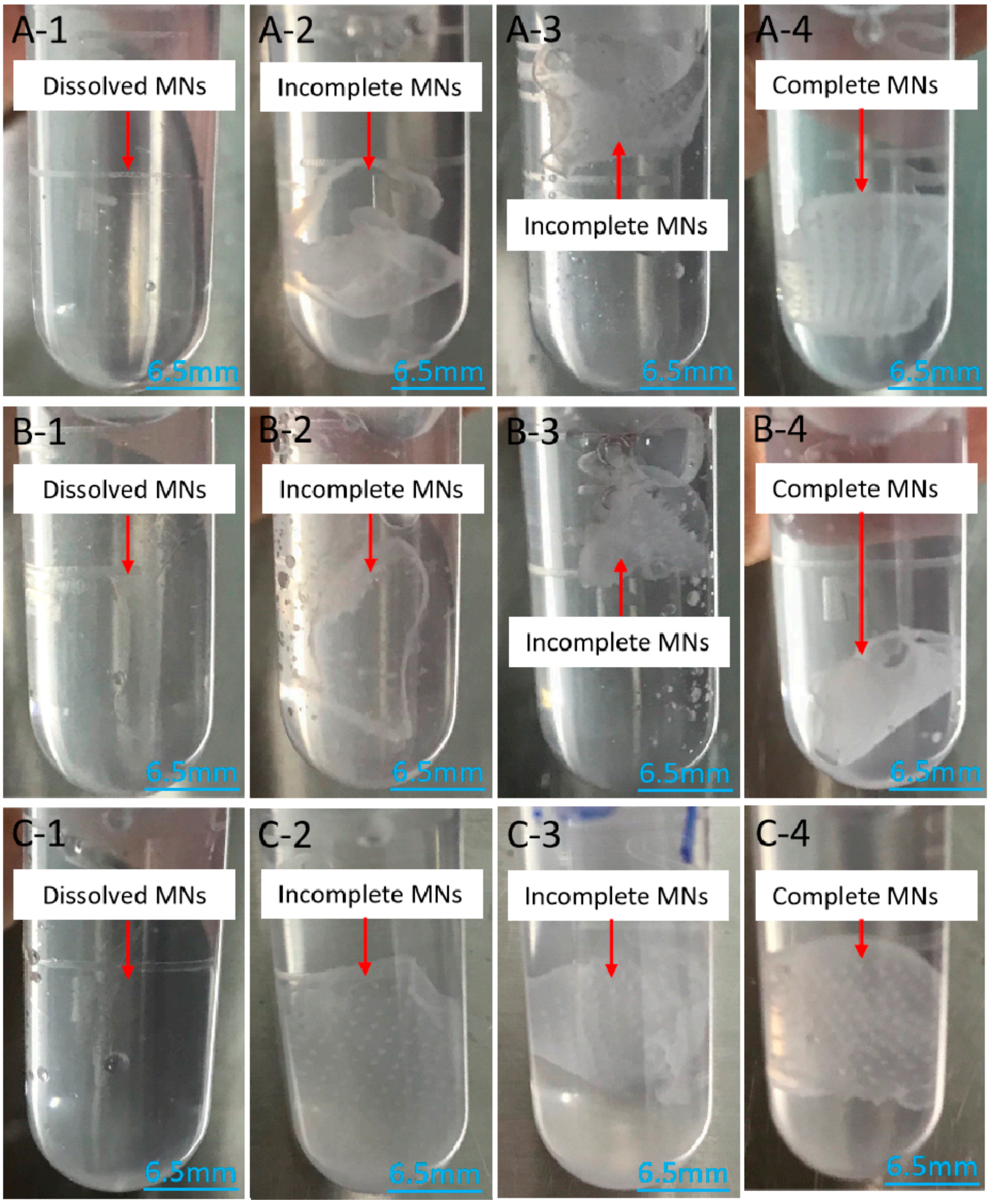

Figure S1: (**A**) Morphological integrity of microneedles after swelling prepared with 10% PVA + 0% Eudragit NM30D (A-1), 10% PVA + 2% Eudragit NM30D (A-2), 10% PVA + 3% Eudragit NM30D (A-3), and 10% PVA + 4% Eudragit NM30D (A-4), respectively; (**B**) Morphological integrity of microneedles after swelling prepared with 12% PVA + 0% Eudragit NM30D (B-1), 12% PVA + 2% Eudragit NM30D (B-2), 12% PVA + 3% Eudragit NM30D (B-3), and 12% PVA + 4% Eudragit NM30D (B-4), respectively; (**C**) Morphological integrity of microneedles after swelling prepared with 15% PVA + 0% Eudragit NM30D (C-1), 15% PVA + 2% Eudragit NM30D (C-2), 15% PVA + 3% Eudragit NM30D (C-3), and 15% PVA + 4% Eudragit NM30D (C-4), respectively

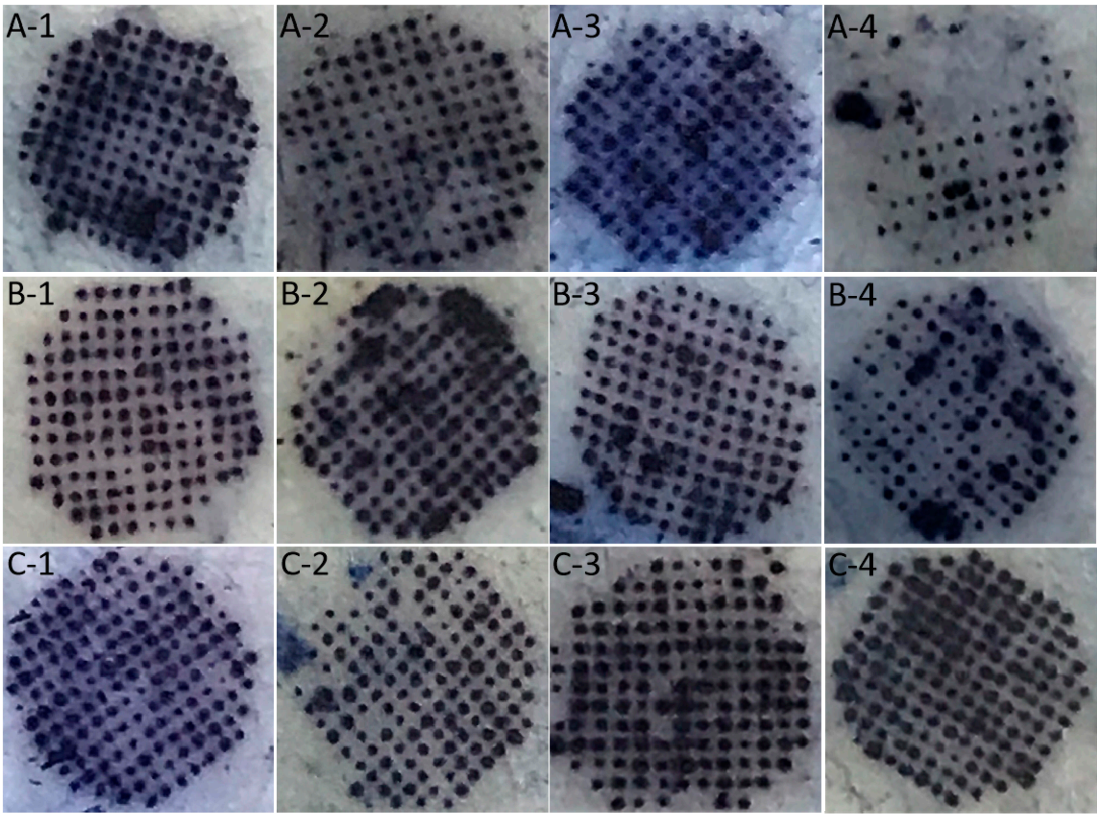

Figure S2: Skin insertion arrays of microneedles prepared with 10% PVA and different proportions of Eudragit NM30D (A-1 to A-4 were 0, 2, 3, 4% Eudragit NM30D, respectively); (**B**) Morphological integrity of microneedles prepared with 12% PVA and different proportions of Eudragit NM30D (B-1 to B-4 were 0, 2, 3, 4% Eudragit NM30D, respectively); (**C**) Morphological integrity of microneedles prepared with 15% PVA and different proportions of Eudragit NM30D (C-1 to C-4 were 0, 2, 3, 4% Eudragit NM30D, respectively).

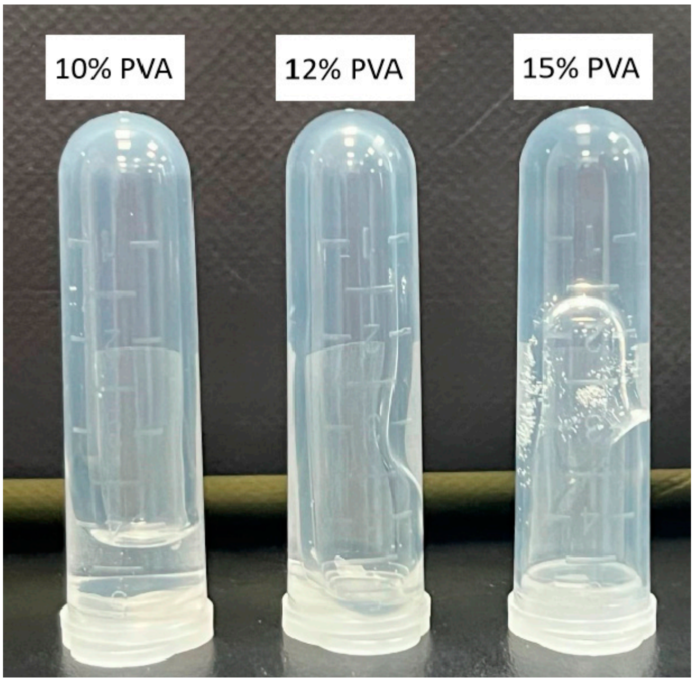

Figure S3: The physical images of PVA solution of 10%, 12% and 15%, respectively.

Table S1. Formulation of MNs composed of PVA and Eudragit NM30D.

Formulation code	PVA (%, w/v)	Eudragit NM30D (%, w/v)
H1	10	0
H2	10	2
H3	10	3
H4	10	4
H5	12	0
H6	12	2
H7	12	3
H8	12	4
H9	15	0
H10	15	2
H11	15	3
H12	15	4
